# Anti-SmD1 antibodies are associated with renal disorder, seizures, and pulmonary arterial hypertension in Chinese patients with active SLE

**DOI:** 10.1038/s41598-017-08099-3

**Published:** 2017-08-08

**Authors:** Chaojun Hu, Mengtao Li, Jian Liu, Junyan Qian, Dong Xu, Shulan Zhang, Ping Li, Jiulang Zhao, Xinping Tian, Xiaofeng Zeng

**Affiliations:** 1 Department of Rheumatology, Peking Union Medical College Hospital, Peking Union Medical College & Chinese Academy of Medical Sciences, Key Laboratory of Rheumatology and Clinical Immunology, Ministry of Education, No. 1 Shuaifuyuan, Beijing, 100730 China; 2 Department of Rheumatology, Aerospace Center Hospital, Aerospace Clinical Medical College, Peking University, No. 15 Yuquan Road, Haidian District, Beijing, 100049 China

## Abstract

Detection of autoantibodies in systemic lupus erythematosus (SLE) plays an important role in timely diagnosis and earlier treatment of SLE. In this study, we used a SmD1 polypeptide-based ELISA to determine anti-SmD1 antibody in 269 SLE, including100 naïve (had not been treated with steroids or immunosuppressants at study inception) SLE patients and 169 non-naive SLE patients; 233 controls with other rheumatic diseases (RDC) (70 RA, 40 AS, 73SSc, and 50 SS), and 110 healthy controls (HC) group. The positive rate of anti-SmD1 among all SLE patients was 60.97%, higher than that in the RDC group (13.30%, *P* = 0.000) or the HC group (9.09%, *P* = 0.000). The positive rate of anti-SmD1 in non-naive SLE patients was higher than that for anti-dsDNA antibodies (44.97%, *P* = 0.03). Positivity for anti-SmD1 only was found in 14.00% of naive SLE patients and 16.00% of non-naive SLE patients. In naive SLE patients, the serum concentration of anti-SmD1 was lower after treatment than before treatment (*P* = 0.039). Active SLE patients positive for anti-SmD1 were more likely to have malar rash, rash, nonscarring alopecia, PAH and hypocomplementemia. High positivity for anti-SmD1 only in patients with SLE indicated the importance and necessity of detection of anti-SmD1 in patients with SLE.

## Introduction

Systemic lupus erythematosus (SLE) is a complicated, multi-systemic autoimmune disease with unknown pathogenesis and with the largest number of detectable autoantibodies^1^. The complexity of clinical features that involve almost every organ of the human body is matched by the diversity of different antibodies found in SLE patients. It has been proven that autoantibodies contribute to the pathologic changes and development of SLE. The presence of autoantibodies can be detected many years before the diagnosis of SLE^2^. Furthermore, the appearance of autoantibodies in patients with SLE tends to follow a predictable course, with a progressive accumulation of specific autoantibodies before the onset of SLE, while patients are still asymptomatic. In the past decade, patients’ quality of life and survival continue to be improved along with progress in timely, appropriate diagnosis and earlier treatment of SLE^3^, for which the detection of autoantibodies plays an important role. Presently, there are more than 180 different antibodies found in SLE patients^4^, some of which are used as disease diagnostic markers while others are used as disease activity markers. Among those autoantibodies, the anti-dsDNA antibodies and anti-Sm antibodies are the most important autoantibodies in SLE. Since anti-dsDNA antibodies not only serve as a specific diagnostic marker but also a reliable index of disease activity in SLE, they have been included in both the American College of Rheumatology criteria for classification of SLE^5^ and the Systemic Lupus International Collaborating Clinics Classification Criteria for SLE^6^. Furthermore, as laboratory abnormalities, especially positivity for autoantibodies, have become increasingly important in the Classification Criteria for SLE, there has been more effort devoted to the study of autoantibodies.

Small nuclear ribonucleoproteins (snRNPs), which are also well known as Smith (Sm) antigen, represent a very important autoantigen in SLE. Anti-Sm antibody was first detected in sera of SLE patients in 1966^7^. The following decades of research on anti-Sm antibodies indicated that the anti-Sm antibodies are SLE-specific autoantibodies, which are only present in approximately 10–30% of SLE patients and rarely present in sera from patients with other rheumatic diseases. Anti-Sm antibodies also were demonstrated to be associated with acute confusional state, nephritis, and hemophagocytosis in patients with SLE^8–11^. Further studies have indicated that Sm antigen is a part of the spliceosomal complex and is composed of at least nine different polypeptides with molecular weights ranging from 9–29.5 kDa, including (B (B1, 28 kDa), B′ (B2, 29 kDa), N (B3, 29.5 kDa), D1 (16 kDa), D2 (16.5 kDa), D3 (18 kDa), E (12 kDa), F (11 kDa), and G (9 kDa)). The nine different polypeptides of Sm antigen are just the core proteins of the U1, U2, U4, U5, U6, U7, U11, and U12 snRNPs, respectively. Although each of the different polypeptides of Sm have antigenicity against the anti-Sm antibodies, the major target Sm antigens are the B polypeptides and the D1 polypeptide^12^. It is known that the conformational epitopes of an antigen affect the binding of antigen to antibody, which can further influence the sensitivity and specificity of various detection methods established based on this antigen.

Given the low positive rate of anti-Sm antibodies in SLE, these antibodies do not seem to be perfect in clinical use, and it has been demonstrated that the SmD1 protein shares conformational epitopes that appear to not be accessible in the Sm antigen (spliceosomal complex)^13^. Theoretically, the sensitivity of methods established based on SmD1 protein is better than that of those established based on Sm antigen. In this study, we applied a SmD1 (Peptide aa 83–119) polypeptide-based enzyme-linked immunosorbent assay (ELISA) to detect anti-SmD1 antibodies in patients with SLE, patients with other autoimmune diseases, and healthy volunteers to explore the positivity and specificity of anti-SmD1 antibodies in naive SLE patients (those not previously treated with steroids or immunesuppressants at the inception of the study) and non-naive SLE patients as well as the associations between anti-SmD1 antibodies and clinical features of SLE.

## Results

### Demographic data of SLE patients

The 269 SLE patients included in this study were divided into the naive SLE group and non-naive SLE group. The mean SLEDAI did not differ significantly between naive (9.02 ± 8.01) and non-naive (10.60 ± 8.45) SLE patients, but the incidences of arthritis, renal disorder, neurologic disorder, and abnormal antinuclear antibody in non-naive SLE patients were higher than those in naive SLE patients. The demographic characteristics of the SLE patients included in this study are presented in Table 1.

**Table 1:** Demographic and clinical characteristics of SLE patients.

Demographic variables	Naive SLE	Non-naive SLE	*χ* ^2^/*t*	*P*
Cases	100	169		
Gender (M/F)	4/96	17/152	3.20	0.073
Age (years)^*^	31.47 ± 12.2	32.3 ± 10.3	0.76	0.451
**Clinical features**				
SLEDAI^*^	9.02 ± 8.01	10.60 ± 8.45	1.52	0.131
Malar rash	46 (46.00%)	91 (53.85%)	1.55	0.213
Discoid rash	8 (8.00%)	16 (9.47%)	0.17	0.683
Photosensitivity	28 (28.00%)	50 (29.59%)	0.08	0.782
Oral ulcers	21 (21.00%)	50 (29.59%)	1.49	0.222
Arthritis	33 (33.00%)	86 (50.89%)	8.15	0.004
Serositis	15 (15.00%)	36 (21.30%)	1.62	0.203
Renal disorder	35 (35.00%)	81 (47.93%)	4.28	0.039
Neurologic disorder	3 (3.00%)	44 (26.04%)	21.55	0.000
Hematologic disorder	56 (56.00%)	97 (57.40%)	0.05	0.823
Immunologic disorder	76 (76.00%)	140 (82.84%)	1.86	0.173
Antinuclear antibody	84 (84.00%)	157 (92.90%)	5.34	0.021

^*^Statistical analysis of age and SLEDAI adopted independent-sample test.

### Anti-SmD1, anti-Sm, and anti-dsDNA antibodies in different patients

**Figure 1:**
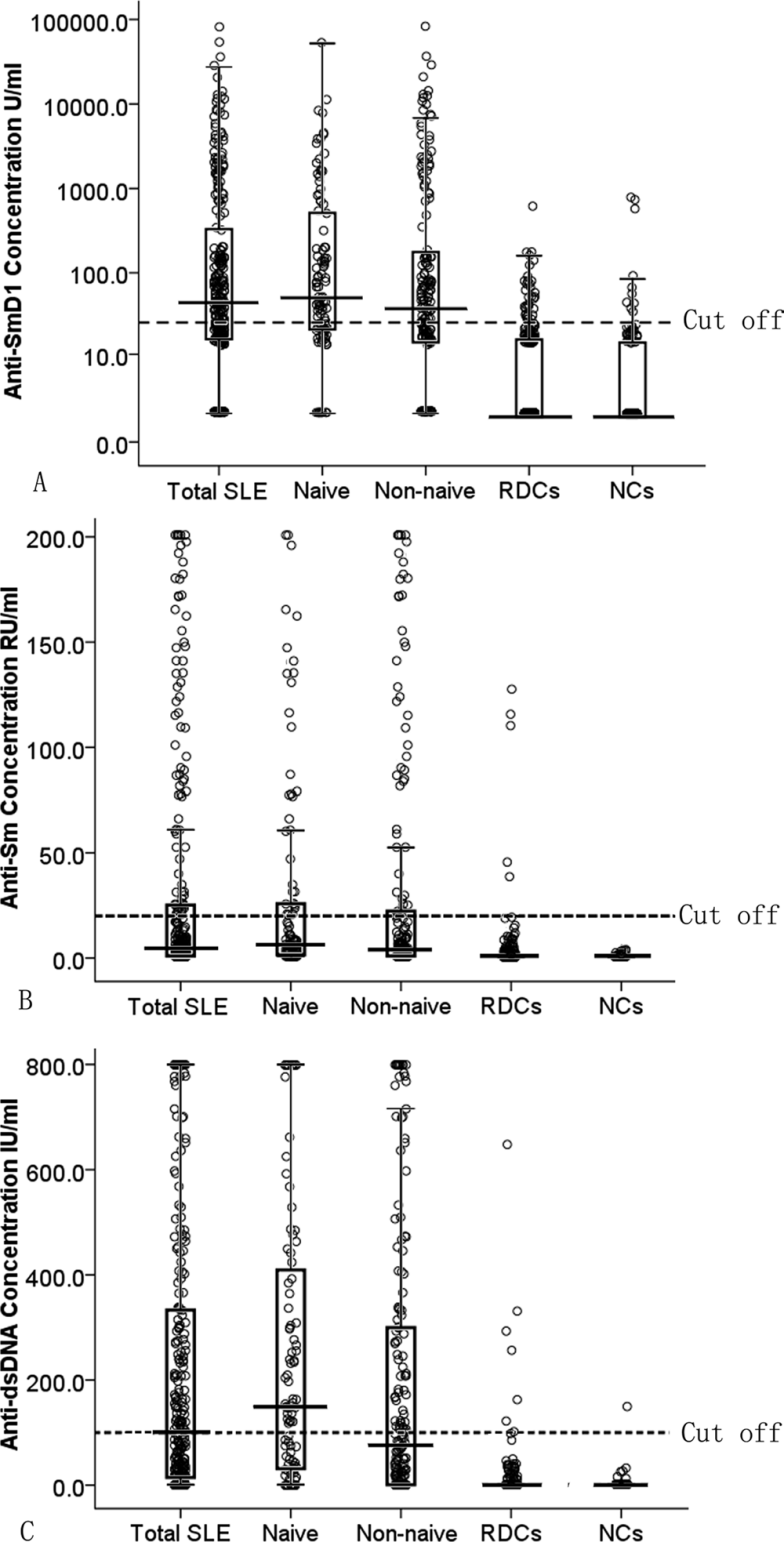
Quantification of serum anti-SmD1, anti-Sm, and anti-dsDNA antibody concentrations in SLE patients, rheumatic disease control and healthy control.

We found that the concentration of anti-SmD1 autoantibodies was significantly higher in the SLE group (1630.6 ± 6686.9 U/ml) than in the RDC group (15.3 ± 43.3 U/ml; *P* = 0.000) or the HC group (25.1 ± 105.7 U/ml; *P* = 0.000). The positive rate of anti-SmD1 in 269 patients with SLE was 60.97%, which was significantly higher than that in the RDC group(13.30%, *P* = 0.000) or the HC group (9.09%, *P* = 0.000). The mean concentration of anti-Sm antibodies for the SLE group was 31.66 ± 55.3 RU/ml, and the positive rate of anti-Sm in SLE patients was28.25%, which was significantly higher than that in the RDC group (2.15%, *P* = 0.000) or the HC group (0.00%, *P* = 0.000). Patients with SLE also had a significantly greater mean concentration of anti-dsDNA antibodies (217.8 ± 261.9 IU/ml) than that those in the RDC group (13.0 ± 56.3 IU/ml; *P* = 0.000) or HC group (3.3 ± 14.8 IU/ml; *P* = 0.000). Of the 269 patients with SLE, 135 (50.19%) were positive for anti-dsDNA antibodies, compared with 7of 233 (3.00%) patients in the RDC group and none of the 110 patients (0.00%) in the HC group. The positive rates of anti-SmD1 in naive and non-naive SLE patients were 68.00% and 56.80%, respectively, which was higher than that for anti-Sm (32.00%, *χ* ^2^ = 25.92, *P* = 0.000; 26.04%, *χ* ^2^ = 32.97, *P* = 0.000). The positive rate of anti-SmD1 in non-naive SLE was higher than that of anti-dsDNA antibodies (44.97%, *χ* ^2^ = 4.74, *P* = 0.03), but this difference was not been found in naive SLE patients. Quantification of serum anti-SmD1, anti-Sm, and anti-dsDNA antibody concentrations in SLE patitents, rheumatic disease control and healthy control are shown in Fig. 1.

### Performance of the anti-SmD1, anti-Sm, and anti-dsDNA antibodies

For the 100 naive SLE patients, the diagnostic performance of the antibodies was evaluated for sensitivity, specificity, and accuracy and found to be 68.00%, 88.05%, and 83.52% for anti-SmD1, respectively; 32.00%, 98.54%, and 83.52%, respectively, for anti-Sm; and 59.00%, 97.28%, and 89.16%, respectively, for anti-dsDNA. The sensitivity of anti-SmD1 was significantly higher than that of anti-Sm (*χ* ^2^ = 25.92, *P* = 0.000), and the diagnostic sensitivity of combined detection of anti-SmD1 and anti-dsDNA was higher than that for a single assay of anti-dsDNA (*χ* ^2^ = 9.35, *P* = 0.002). Compared with the single assay for anti-dsDNA, the combined detection of anti-Sm and anti-dsDNA did not contribute to improvements in the diagnostic sensitivity, specificity and accuracy for SLE. These results are summarized in Table 2.

**Table 2:** Diagnostic performance of the anti-SmD1, anti-Sm and anti-dsDNA antibodies.

Autoantibody	Sensitivity	Specificity	Accuracy
Anti-SmD1	68.00%	88.05%	83.52%
Anti-Sm	32.00%	98.54%	83.52%
*χ*_a_^2^	25.92	30.20	0.00
*P*_a_	0.000	0.000	1.000
Anti-dsDNA	59.00%	97.96%	89.16%
*χ*_b_^2^	1.75	25.90	5.98
*P*_b_	0.186	0.000	0.014
Anti-SmD1 or Anti-dsDNA	79%	85.71%	84.20%
*χ*_c_^2^	9.35	34.30	4.73
*P*_c_	0.002	0.000	0.030
Anti-Sm or Anti-dsDNA	67%	96.50%	89.84%
*χ*_d_^2^	1.37	1.35	0.11
*P*_d_	0.240	0.245	0.742

Note: a, analyses between anti-SmD1 and anti-Sm; b, analyses between anti-SmD1 and anti-dsDNA; c, analyses between anti-SmD1oranti-dsDNAand anti-dsDNA; d, analyses between anti-Sm or anti-dsDNA and anti-dsDNA.

### Cross-positivity analyses for the anti-SmD1, anti-Sm and anti-dsDNA autoantibodies in patients with SLE

**Figure 2:**
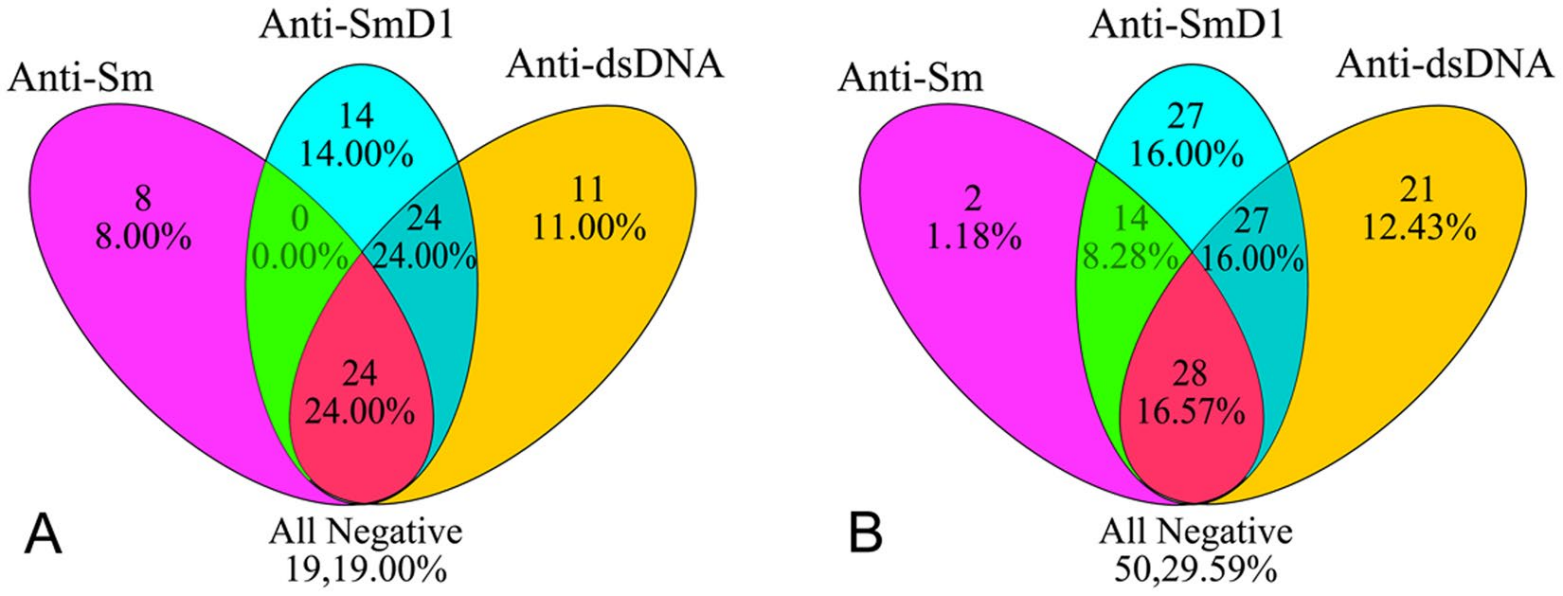
Cross-positivity for anti-SmD1, anti-Sm, and anti-dsDNA antibodies in SLE patients. (**A**) In100 naive SLE patients; (**B**) in169 non- naive SLE patients.

Among the 100 naive SLE patients and 169 non-naive SLE patients, 24 (24.0%) and 28 (16.6%) were positive for all three tested autoantibodies. Positivity for anti-SmD1 only was 14.0% in the naive SLE group and 16.0% in non-naive SLE group. Positivity for anti-Sm only was 8.0% in the naive SLE group, which was higher than 1.2% in non-naive SLE (*χ* ^2^ = 6.36, *P* = 0.012), which indicates the positivity of anti-Sm was affected more readily by treatment than was the presence of anti-SmD1. Cross-positivity for anti-Sm and anti-dsDNA was not seen in either naive or non-naive SLE. Of the 169 non-naive SLE patients, the positivity for anti-SmD1 only (16.0%) was higher than that for anti-Sm only (1.2%, *χ* ^2^ = 23.57, *P* = 0.000). The results of cross-positivity for anti-SmD1, anti-Sm, and anti-dsDNA are shown in Fig. 2.

### Concentration of anti-SmD1 in eight naive SLE patients before and after treatment

**Figure 3:**
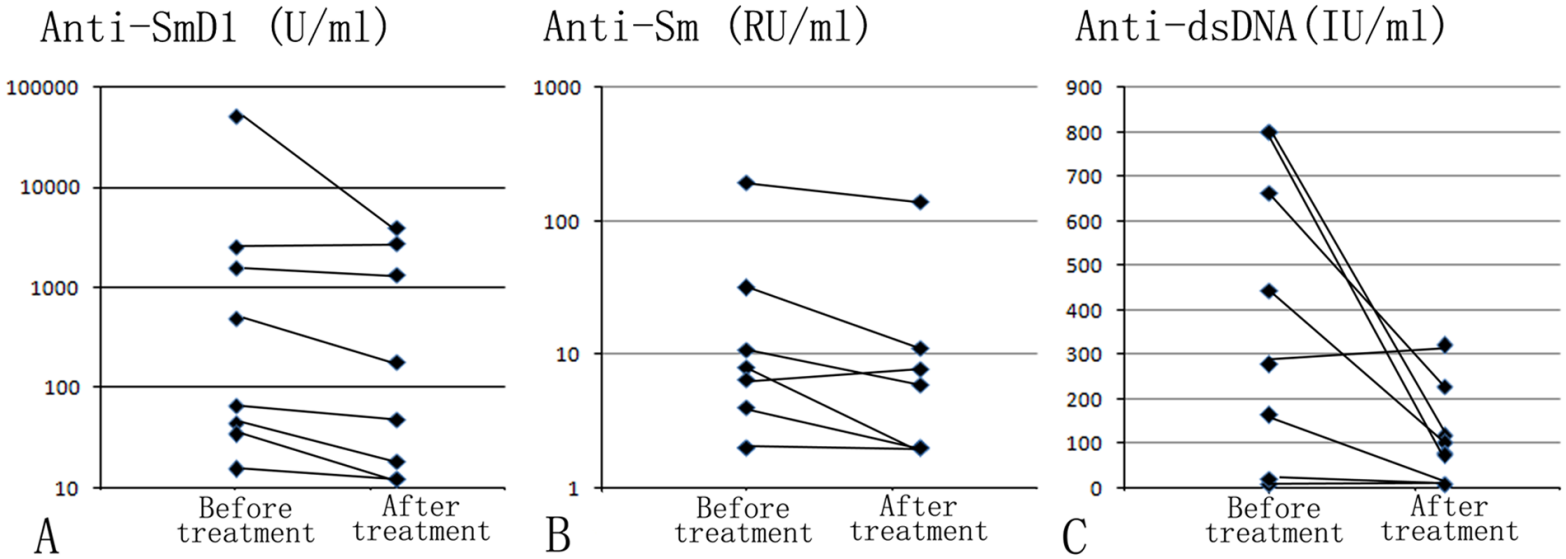
Concentrations of anti-SmD1, anti-Sm and anti-dsDNA in eight naive SLE patients before and after treatment.

Among the 100 naive SLE patients, we detected anti-SmD1, anti-Sm, and anti-dsDNA antibodies in the serum of eight patients before and after treatment. After conducting the logarithm transformation of anti-SmD1, anti-Sm and anti-dsDNA concentrations, paired t-tests were used to analyze the anti-SmD1, anti-Sm, and anti-dsDNA levels in the serum of patients before and after treatment. The concentrations of anti-SmD1, anti-Sm, and anti-dsDNA in the serum of treated naive SLE patients was significantly lower than that in the serum before treatment (*t* = 2.54, *P* = 0.039; *t* = 2.49, *P* = 0.042; *t* = 3, 24, *P* = 0.014, respectively). The results of the eight individuals were shown in Fig. 3.

### Associations between SLE-related clinical features of 183 patients with active SLE and presence of anti-SmD1

Among various clinical manifestations presented by our patients (Table 3), the presence of anti-SmD1 antibodies was significantly associated with malar rash (*χ* ^2^ = 8.56, *P* = 0.003), rash (*χ* ^2^ = 23.72, *P* = 0.000), nonscarring alopecia (*χ* ^2^ = 11.25, *P* = 0.001), renal disorder (*χ* ^2^ = 7.90, *P* = 0.005), proteinuria (*χ* ^2^ = 5.34, *P* = 0.021), seizures (*χ* ^2^ = 13.06, *P* = 0.000), pulmonary arterial hypertension (PAH) (*P* = 0.014), and hypocomplementemia (*χ* ^2^ = 8.03, *P* = 0.005). These results are summarized in Table 3.

**Table 3:** Associations between SLE-related clinical features of 183 patients with active SLE and anti-SmD1 levels.

Clinical features	Anti-SmD1		*χ* ^2^	*P*	Clinical features	Anti-SmD1		*χ* ^2^	*P*
	Pos	Neg				Pos	Neg		
Malar rash	71	26	8.56	0.003	Seizures	12	46	13.06	0.000
	45	41				61	64		
Discoid rash	12	8	0.11	0.739	Hematologic disorder	66	41	0.32	0.570
	104	59				50	26		
Rash	71	16	23.72	0.000	Hemolytic anemia	13	9	0.20	0.656
	45	51				103	58		
Photosensitivity	40	19	0.73	0.393	Leukopenia	56	35	0.27	0.605
	76	48				60	32		
Nonscarring alopecia	53	14	11.25	0.001	Pericarditis	23	8	1.88	0.171
	63	53				93	59		
Oral ulcers	32	15	0.60	0.438	Interstitial lung disease	10	4	0.65	0.419
	84	52				16	14		
Arthritis	52	26	0.63	0.427	Pulmonary arterial hypertension	8	0		0.014
	64	41				18	18		
Serositis	24	11	0.50	0.479	Pulmonary infiltration	4	7	2.01	0.157
	92	56				22	11		
Renal disorder	46	41	7.90	0.005	Pleural effusion	4	5	0.39	0.534
	70	26				22	13		
Proteinuria	47	39	5.34	0.021	Immunologic disorder	97	54	0.27	0.604
	69	28				19	13		
Hematuria	34	27	2.31	0.129	Hypocomplementemia	96	43	8.03	0.005
	82	40				20	24		
Neurologic disorder	25	18	0.67	0.414	Antinuclear antibody	103	61	0.23	0.630
	91	49				13	6		

## Discussion

SLE is one of the most complicated diseases among autoimmune diseases, with systemic autoimmune damage. Patients with SLE often present with dysfunction of the CNS, kidneys, skin, hematopoietic organs, and other organs. In this study, we applied the SmD1 polypeptide-based ELISA to detect anti-SmD1 antibodies in a large cohort of patients with SLE, patients with other autoimmune diseases, and healthy volunteers. As autoantibodies in the serum were greatly affected by the treatment, different from other studies, the patients with SLE included in our study were divided into naive SLE and non-naive SLE groups. First, we found a high positivity of anti-SmD1 in patients with SLE, 68.00% in naive and 56.80% in non-naive SLE patients, respectively, and the results were consistent with previous studies^13^. It is worth to note that in our study, the positive rates of anti-SmD1 in naive, non-naive and unselected total SLE patients were 68.00%, 56.80% and 60.97% respectively, which indicate that the positive rate of anti-SmD1 in patients with SLE was fluctuated with the different patients recruited. Second, we also identified the relationships between anti-SmD1 autoantibodies and SLE-related clinical features in active SLE, including malar rash, nonscarring alopecia, renal disorder, proteinuria, seizures, PAH, hypocomplementemia, and so on.

Compared to those in the RDC and HC groups, the concentration and positivity rate of anti-SmD1 autoantibodies were significantly higher in the SLE group. Both in the naive SLE and non-naive SLE patients, the positive rate of anti-SmD1 autoantibodies was significantly higher than the positive rate of anti-Sm autoantibodies. It is worth noting that the positive rate of anti-SmD1 antibodies (56.80%) in non-naive SLE patients was significantly higher than that of anti-dsDNA antibodies (44.97%), but this difference was not been found in naive SLE patients (68.00% and 59.00%), which indicated the importance of detection of anti-SmD1 in non-naive SLE. Among the 100 naive SLE patients, the sensitivity of anti-SmD1was 68.00%, which was almost the same as that of anti-dsDNA (59.00%) and was significantly higher than that of anti-Sm. We observed that compared to the single assay of anti-dsDNA, the combination of anti-Sm and anti-dsDNA did not improve the diagnostic sensitivity, specificity and accuracy for SLE, while the combination of anti-SmD1 and anti-dsDNA antibodies outperformed in terms of the sensitivity of anti-SmD1 or anti-dsDNA antibodies alone.

Beyond previous studies, we also evaluated cross-positivity for the anti-SmD1, anti-Sm and anti-dsDNA autoantibodies in patients with SLE. The results indicated that 19.00% and 29.59% of naive SLE and non-naive SLE patients had no anti-SmD1, anti-Sm and anti-dsDNA autoantibodies, respectively, whereas 24.00% of 100 naive SLE patients and 16.57% of the 169 non-naive SLE were positive for all three tested autoantibodies. Positivity for anti-Sm only was found in 8.0% of naive SLE patients, and this rate was higher than the 1.2% in non-naive SLE patients. However, this difference was not found for anti-SmD1 only or anti-dsDNA only. High positivity for anti-SmD1 only among patients with SLE (14.0% in naive SLE and 16.0% in non-naive SLE) indicated the importance and necessity of detectinganti-SmD1 in patients with SLE, especially in anti-dsDNA negative SLE patients.

We performed an analysis of associations between anti-SmD1 and SLE-related clinical features in 183 patients with active SLE. We found that anti-SmD1 positivity was associated with malar rash, rash, nonscarring alopecia, PAH, hypocomplementemia, seizures, and renal disorders such as proteinuria. SLE patients positive for anti-SmD1 were more likely to have malar rash, nonscarring alopecia, PAH, and hypocomplementemia, but were not prone to have seizures or renal disorders such as proteinuria. PAH, although it has received little attention, is a severe complication with high morbidity and mortality rates and is the 3rd leading cause of death in Chinese SLE patients^14^. Our previous research has shown that anti-Sm was more frequently present in patients with SLE and PAH^15^. In this study, the results indicated that SLE patients positive for anti-SmD1 were also prone to have PAH. Seizures and lupus nephritis are severe manifestations of SLE and affect up to 32–68% of patients in the course of the disease. Previous studies have demonstrated that the anti-Sm autoimmune response is a polyclonal humoral immune response against protein components of snRNP particles, and it is found in about 30% of SLE patients^16^. Anti-Sm antibodies have demonstrated greater effects on the grey matter density (GMD)^17^, and the association between anti-Sm antibodies and the GMD reduction suggests a possible diagnostic and prognostic value of anti-Sm antibodies in determining CNS involvement in SLE. Moreover, a correlation between the presence of anti-Sm antibodies in serum and central NPSLE has been observed^18^. Anti-Sm antibody was considered to be independently associated with a higher incidence of seizure^19^ and lupus nephritis^9^, but, interestingly, in our cohort, we observed that SLE patients positive for anti-SmD1 were not likely to have seizures or renal disorders such as proteinuria. Regardless, the anti-SmD1 antibody positivity was significantly related to many clinical features. However, there were no significant associations between positivity for anti-SmD1 antibodies and other clinical characteristics of SLE such as oral ulcers, arthritis, serositis, or hematologic disorder.

## Conclusions

In conclusion, this is a comprehensive report on the significance of the presence of anti-SmD1 antibodies in the serum of Chinese patients with SLE, especially in the naive SLE patients. Our study indicated that there is important clinical value of anti-SmD1 Abs in the SLE as the positivity for anti-SmD1 only was found in 14.00% of naive SLE patients and 16.00% of non-naive SLE patients, and the presence of anti-SmD1 antibodies was significantly associated with malar rash, rash, nonscarring alopecia, renal disorder, proteinuria, seizures, PAH, and hypocomplementemia. From a clinical point of view, these data support the usefulness of anti-SmD1 as an additional serological autoantibody for the diagnosis of SLE and a predictive marker for some SLE-related manifestations. However, additional longitudinal, multicenter studies are needed to validate the diagnostic and/or prognostic role of anti-SmD1 in a large cohort of patients with SLE.

## Materials and Methods

### Ethical approval

This study was approved by the Medical Ethics Committee of Peking Union Medical College Hospital (PUMCH). The methods were carried out in accordance with the principles stated in the Declaration of Helsinki. Informed consent was obtained from each patient.

### Patients and samples

For this study, we used serum samples from the Biobank of the Chinese Rheumatism Data Center (CRDC), collected between Mar 2011 and Feb 2016. All 269 SLE patients recruited for this study including100 naive SLE patients (had not been treated with steroids or immunosuppressants at study inception, 31.2 ± 12.6 years, 96 female) and 169 non-naive SLE patients (32.3 ± 10.3 years, 152 female), were from the PUMCH center and registered in the Chinese Systemic Lupus Erythematosus (SLE) Treatment and Research (CSTAR) group registry database^20^, which includes 300 high-ranking rheumatology centers, covering 30 provinces in China. All SLE patients fulfilled the 1997revised American College of Rheumatology classification criteria^21^. The rheumatic disease control (RDC) group comprised 233 patients with other rheumatic diseases, including 70 with rheumatoid arthritis (RA, 46.0 ± 12.9 years, 66 female), fulfilling the 2010 criteria of the American College of Rheumatology and European League Against Rheumatism^22^; 40 with ankylosing spondylitis (AS, 36.3 ± 13.5years, 6 female) fulfilling the 1984modified New York criteria^23^;73 with systemic sclerosis (SSc, 44.7 ± 10.4years, 67 female) fulfilling the American College of Rheumatology criteria for the classification of SSc^24^; and 50 with Sjogren’s syndrome (SS, 50.0 ± 15.8years, 48 female) fulfilling the American College of Rheumatology classification criteria for Sjogren’s syndrome^25^. Also, 110 healthy volunteers were also recruited as healthy controls (HC, 37.0 ± 8.4years, 70 female). Four-milliliter blood samples were collected using a BD vacutainer without anticoagulants and clotted at room temperature for up to 1 h, before being centrifuged at 4 °C for 5 min at 3,000 rpm. The serum was then allocated and stored at −80 °C. No sample was subjected to more than one freeze–thaw cycle before analysis.

### Detection of serum anti-SmD1, anti-Sm, and anti-dsDNA antibodies by ELISA

Anti-SmD1, anti-Sm, and anti-dsDNA antibodies in serum were detected following standard protocols of ELISA reagent kits (Anti-SmD1 ELISA kit purchased from HUMAN, Germany; Anti-Sm and anti-dsDNA ELISA kits purchased from EUROIMMUNE, Germany). Serum samples were diluted 1:200 with sample buffer (1:100 for detection of anti-SmD1 antibodies), and 100 μl of each diluted serum sample, reference standard, or negative or positive control solution were added to the designated antigen-coated micro wells. After incubation for 30 minutes (1 hour for anti-SmD1 antibodies) at room temperature, the micro plates were washed with 300 μl washing buffer three times, and 100 μl conjugate was added to each well and incubated for 30 minutes at room temperature. Then the micro plate was washed again with 300 μl washing buffer three times, and 100 μl substrate was added into each well and incubated for 15 minutes (10 minutes for anti-SmD1 antibodies) at room temperature. Then 100 μl stop solution was added to each well, the optical density of all wells was obtained using a microplate reader. All samples with an optical density higher than the cut-off were considered as positive (The cut off value of anti-SmD1, anti-Sm and anti-dsDNA antibodies is 25 U/ml, 20 RU/ml and 100 IU/ml respectively), and the concentration of each antibody type was calculated according to the respective standard curve.

### Statistical analysis

Differences between the SLE and control groups were assessed using the t-test for continuous variables and*χ* ^2^ or Fisher’s exact test for proportions. The concentration of anti-SmD1 in the same naive SLE patient before and after treatment was analyzed using the paired t-test. The associations between anti-SmD1 antibodies and different clinical variables were investigated for patients with SLE using *χ* ^2^ or Fisher’s exact test. All statistical analyses were performed using the SPSS statistical software package for Windows (version 16.0; SPSS Inc., Chicago, IL, USA) and a two-tailed *P*-value of <0.05 was considered significant.
